# Florfenicol-induced Mitochondrial Dysfunction Suppresses Cell Proliferation and Autophagy in Fibroblasts

**DOI:** 10.1038/s41598-017-13860-9

**Published:** 2017-10-19

**Authors:** Dongfang Hu, Shengliang Cao, Guihua Zhang, Yihong Xiao, Sidang Liu, Yingli Shang

**Affiliations:** 1 College of Animal Science and Technology, Shandong Agricultural University, Taian Shandong, 271018 China; 2 Shandong Provincial Key Laboratory of Animal Biotechnology and Disease Control and Prevention, Shandong Agricultural University, Taian Shandong, 271018 China; 3 Shandong Provincial Engineering Technology Research Center of Animal Disease Control and Prevention, Shandong Agricultural University, Taian Shandong, 271018 China

## Abstract

Florfenicol (FLO) is one of the most popular antibiotics used in veterinary clinic and aquaculture. FLO can inhibit both bacterial and mitochondrial protein synthesis. However, the effects of FLO on mitochondrial function and cellular homeostasis remain unclear. Here we show that FLO inhibits expression of mitochondrial DNA-encoded proteins, decreases mitochondrial membrane potential, and promotes generation of reactive oxygen species (ROS) *in vitro*. As a result, activities of mitochondrial respiratory chain complex I and IV and the cellular ATP level are decreased and mitochondrial morphology is damaged. FLO represses cell growth and proliferation by suppression of phosphorylation of p70S6K through AMPK/mTOR/p70S6K pathway. Furthermore, FLO also induces G0/G1 cell cycle arrest via increase of p21 levels through activating ROS/p53/p21 pathway. Moreover, the clearance of damaged mitochondria by autophagy is impaired, leading to cell proliferation inhibition and promotes cell senescence. In addition, FLO-induced upregulation of cytosolic p53 may contribute to mitophagy deficiency via regulation of Parkin recruitment. In summary, our data suggest that florfenicol is an inhibitor of mitochondrial protein synthesis that can induce noticeable cytotoxicity. Thus, these findings can be useful for guiding the proper use of FLO and the development of safe drugs.

## Introduction

Antibiotics are widely used for therapeutic and prophylactic purposes in human and veterinary medicine and also to promote growth and increase feed efficiencies in food producing animals^1^. However, abused or heavy use of antibiotics leads to development of resistance of target pathogens against antibiotics, induced allergic reactions in some hypersensitive individuals, potential compromise of the human intestinal, heart and immune systems^2^. Florfenicol (FLO) is a broad-spectrum antibiotic widely used in veterinary medicine to against bacterial infections^3^ and to promote animal growth, especially in aquaculture^1^. Previous studies have showed that FLO had side effects in multiple organs of various species^3,4^. However, the detail influences and mechanisms of FLO on mitochondrial function and cellular homeostasis are still unclear.

Mitoribosome is believed distinct from eukaryotic ribosome in cytosol and generally resemble the bacterial ribosome^5^. It’s responsible for the synthesis of 13 mitochondrial DNA-encoded proteins that are integral parts of four mitochondrial respiratory chain complexes (I, III, IV and V). FLO can reversibly bind to the large subunit of bacterial ribosomal and mitoribosome and inhibit peptidyl transferase in both prokaryotic organisms and mitochondria, leading to anti-bacteria effects as well as mitochondrial protein synthesis inhibition and mitochondrial dysfunction^6^. As a result, impaired mitochondria provoke energy-generation defects by the inhibition of mitochondrial ATP synthesis. In the cytoplasm, the adenosine monophosphate-activated protein kinase (AMPK) belongs to a family of serine/threonine protein kinase, which sensors cellular stress and regulates cell metabolism and/or proliferation. For instance, AMPK is activated by alteration of AMP/ATP ratio induced by cellular and environmental stress. In addition, the mammalian target of rapamycin (mTOR) pathway is essential for translation initiation and protein synthesis, which could be inhibited by AMPK, resulting in cell growth and proliferation inhibition^7^. Moreover, damaged mitochondria generate harmful reactive oxygen species (ROS) and induce a greater propensity to trigger cell proliferation inhibition^8^ and cellular senescence^9^. Mitochondrial loss is a prominent aspect of cytotoxicity, but the effect of mitochondrial loss on proliferation is not fully understood.

Generally, dysfunctional or damaged mitochondria are removed by a specific autophagic process called mitochondrial autophagy, which is critical for the maintenance of cell homeostasis, because damaged organelles cannot be diluted by cell proliferation^10^. It’s reported that alterations in the autophagic removal of damaged mitochondria intervene in the pathogenesis and process of cardiac aging, neurodegenerative diseases and ischemia or drug-induced tissue injury^11^.

In this study, we investigated the effect of FLO on mitochondrial function, cell proliferation and mitochondrial autophagy, to find out the mechanism of cytotoxicity caused by FLO-induced mitochondrial dysfunction. This study provides new mechanistic insights into drug-induced cytotoxicity, guides proper use of FLO and benefits to the development of safe drugs.

## Results

### FLO inhibits expression of mitochondrial-encoded proteins and impairs mitochondrial function

**Figure 1:**
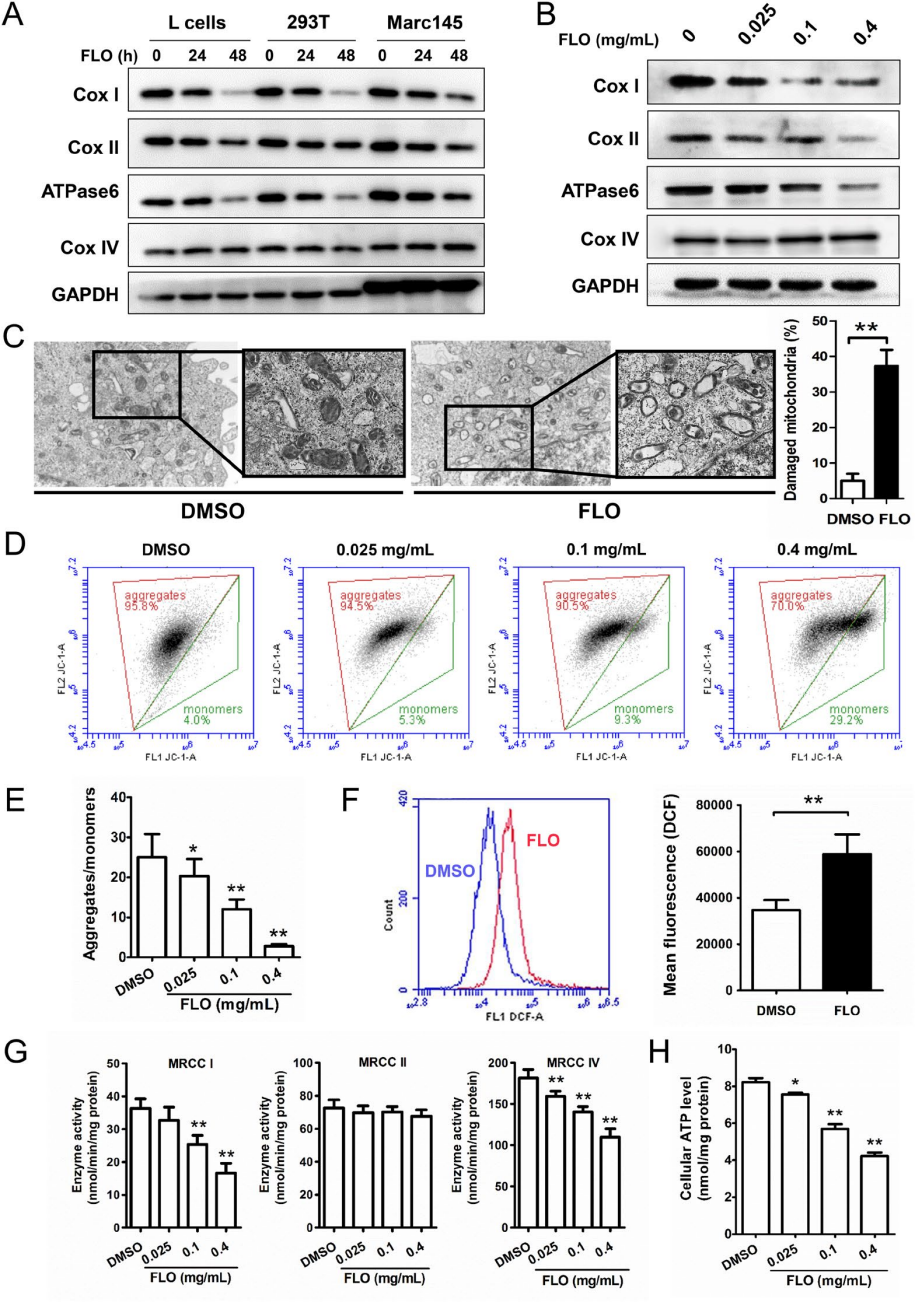
FLO inhibits the expression of mitochondrial-encoded proteins and induces mitochondrial damage and dysfunction. (**A**) Immunoblot analysis of FLO (0.1 mg/mL) on protein levels of Cox I, Cox II, ATPase6 and Cox IV in L cells, 293 T cells and Marc145 cells treated for 24 h or 48 h. (**B**) Immunoblot analysis of multiple doses of FLO on protein levels of Cox I, Cox II, ATPase6 and Cox IV in L cells treated for 24 h. Cropped blots are displayed and full-length blots are included in the Supplementary Information file. (**C**) Electron microscopy pictures that focused on the structure of mitochondria in L cells untreated or treated with FLO (0.1 mg/mL) for 48 h. Data shown are mean ± SD from three independent experiments. **p < 0.01, as determined by LSD multiple comparison tests after one-way ANOVA analysis. (**D**) Flow cytometric analysis of JC-1 aggregates (left) and monomers (right) in L cells treated with multiple doses of FLO for 48 h. (**E**) Ratio of JC-1 aggregates versus monomers in D. (**F**) Flow cytometric analysis of DCF fluorescence in L cells treated with FLO (0.1 mg/mL) for 48 h. The mean fluorescence of DCF represents the level of ROS. (**G**) Enzyme activity analysis of mitochondrial respiratory chain complex I, II and IV of L cells. Data shown are mean ± SD from three independent experiments. (**H**) The influence of FLO treatment for 48 h on ATP biosynthesis in L cells. Data shown are mean ± SD from three independent experiments. *p < 0.05, **p < 0.01, as determined by LSD multiple comparison tests after one-way ANOVA analysis.

Mitochondrial proteins Cox I and Cox II are subunits of respiratory complex IV (cytochrome c oxidase), ATPase6 is a subunit of ATP synthase complexes in the electron transport chain. These three proteins are translated by mitochondrial ribosomes. Cox IV is a component of respiratory complex IV but is encoded by nuclear genome and translated by nuclear ribosomes. To know the effect of FLO on the expression of mitochondrial proteins, we examined the levels of four mitochondrial proteins Cox I, Cox II, ATPase6 and Cox IV in three mammalian cell lines. FLO treatment induced a preferential decrease of Cox I, Cox II and ATPase6 in a time- and dose-dependent manner in all three tested cell lines (Fig. 1A and B). In contrast, FLO did not affect expression of Cox IV (Fig. 1A and B), a mitochondrial protein that is encoded by nuclear genome, suggesting that FLO specifically inhibits mitochondrial DNA (mtDNA)-encoded proteins. We also examined the effect of FLO on mRNA levels of the four mitochondrial proteins and found FLO did not suppresses gene expression of these proteins in these mammalian cell lines. Instead, FLO even increased mRNA levels of Cox I, Cox II and ATPase6 (Supplementary Fig. S1) in these cells. The result is in consistent with a previous report in which inhibition of mitochondrial translation was found to be accompanied by an increase in the expression of mitochondrial-encoded mRNA^12^ and further supporting that FLO inhibits expression of mtDNA-encoded proteins at translational level. To further know the effect of FLO on mitochondrial morphology, FLO-treated and untreated cells were subjected to electron microscopy. The results showed that more damaged mitochondria that characterized by mitochondria swelling, vacuolization and crista fragmentation were found in FLO-treated cells rather than in untreated cells (Fig. 1C), demonstrating that FLO is an inducer of mitochondrial damage.

The mitochondrial respiratory chain generates an electrochemical proton gradient that establishes the mitochondrial membrane potential used to drive ATP generation by complex V. Given FLO induces mitochondrial damage, we therefore examined the effect of FLO on the mitochondrial functions. Upon FLO treatment, L cells showed a decreased mitochondrial membrane potential (MMP) after FLO treatment (Fig. 1D and E). In addition, FLO treatment significantly promotes the generation of reactive oxygen species (ROS), a by-product of mitochondrial respiratory chain in L cells (Fig. 1F). These data suggest that FLO decreases MMP and elevates ROS production of mitochondria, which are characteristics of mitochondrial dysfunction.

We next asked whether a similar exposure to FLO would affect the enzymatic activity of respiratory complexes I and IV that contain proteins translated on mitoribosomes, and respiratory chain complex II, which has no mitochondrial encoded subunits in its substructure^13^. FLO significantly decreased the enzyme activity of respiratory complexed I and IV while had negligible effect on the enzyme activity of the complex II (Fig. 1G), indicating that FLO specifically suppresses activities of enzymes that contain subunits translated by mitoribosomes. As a consequence, levels of ATP, which is the important indicator of efficient mitochondrial respiratory function, were significantly decreased after FLO treatment (Fig. 1H). Together, these data suggest that FLO inhibits expression of mitochondrial-encoded proteins and reduces activities of mitochondrial respiratory chain complexes, leading to mitochondrial dysfunction.

### Mitochondrial dysfunction contributes to FLO-induced cell proliferation inhibition

**Figure 2:**
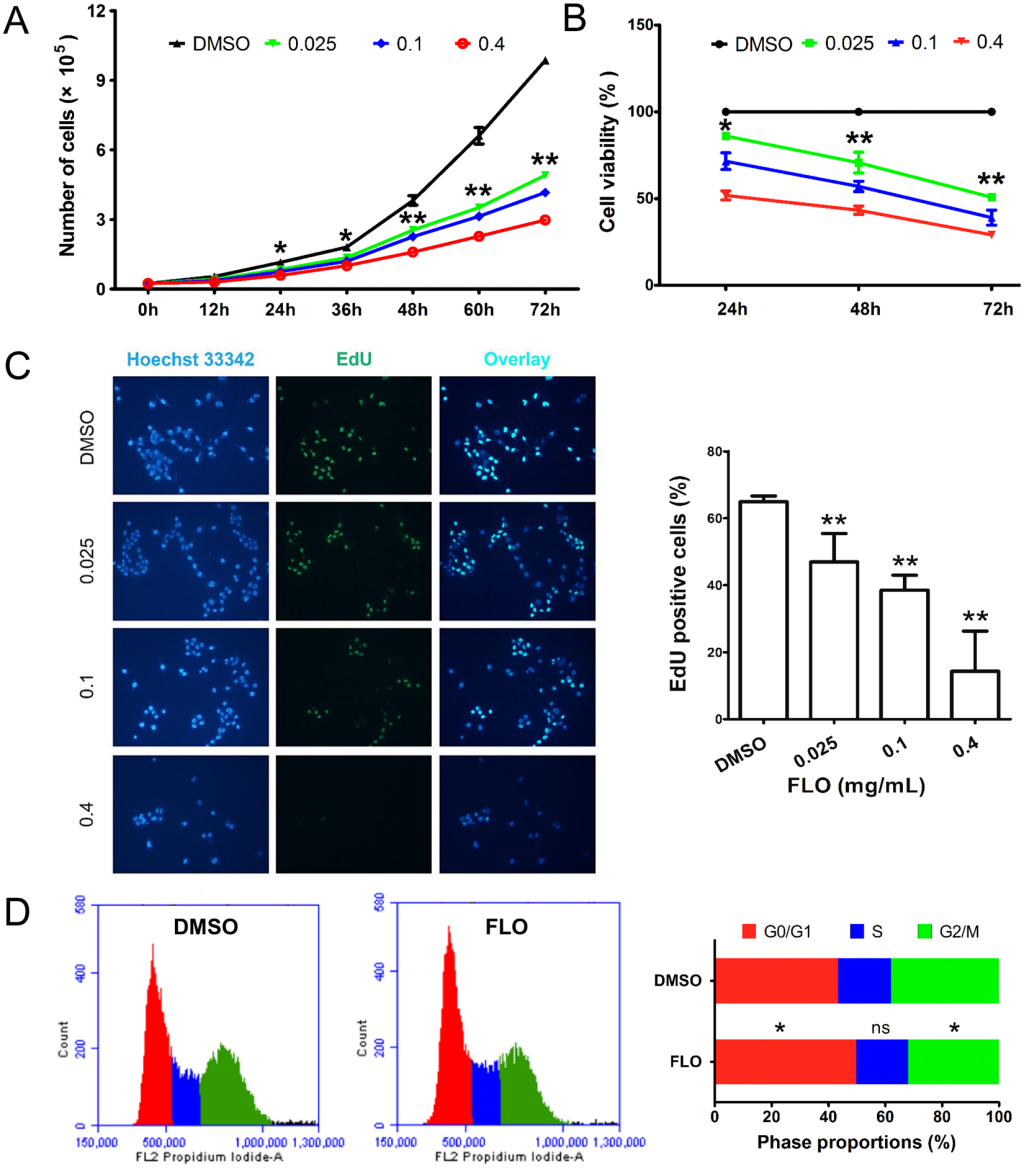
FLO decreases cell viability and induces G0/G1 cell cycle arrest in L cells. (**A**) Growth curve of FLO-treated L cells. The results were expressed as the average cell numbers from three independent experiments. The significance levels between FLO (0.025 mg/mL) group and the control group are shown. (**B**) L cell viability in response to FLO-treatment as detected by CCK-8 method. The results were expressed as the average cell viabilities from three independent experiments. The significance levels between FLO (0.025 mg/mL) group and the control group are shown. (**C**) EdU straining shows the proliferative L cells. EdU positive cells were counted at least 5 randomly selected fields. Each bar represented the mean ± SD of three independent experiments. (**D**) Flow cytometric analysis of cell cycle distribution of L cells treated with FLO (0.1 mg/mL) for 48 h. Data shown are mean ± SD from three independent experiments. *p < 0.05, **p < 0.01, as determined by LSD multiple comparison tests after one-way ANOVA analysis.

To explore the influence of FLO-induced mitochondrial damage on cell survival and proliferation, L cells were incubated with multiple doses of FLO for various hours. As expected, L cells in FLO-containing medium grew much slower than cells in normal medium, indicating that FLO suppresses cell growth (Fig. 2A). Moreover, cell viabilities measured by a CCK-8 assay showed that FLO inhibited cell proliferation in a dose- and time-dependent manner (Fig. 2B). EdU, a rather new thymidine analog that can be incorporated into DNA during active DNA synthesis, has recently been suggested to be a novel highly valid dye for labeling of cells in the S-phase and subsequent tracing of their proliferation ability. We found FLO-treatment significantly reduced the percentage of EdU-positive cells compared to control cells that treated with the solvent DMSO (Fig. 2C), indicating that FLO likely inhibits cell cycle. These observation was further confirmed by flow cytometry post propidium iodide staining, which showed that cell proportion distributed in G0/G1 phase was significantly higher in FLO-treated cells compared to untreated cells (Fig. 2D and Supplementary Fig. S2), demonstrating that FLO treatment induced cell cycle arrest in G0/G1 phase. Altogether, these data suggest that FLO treatment suppresses cell proliferation and induces cell cycle arrest.

**Figure 3:**
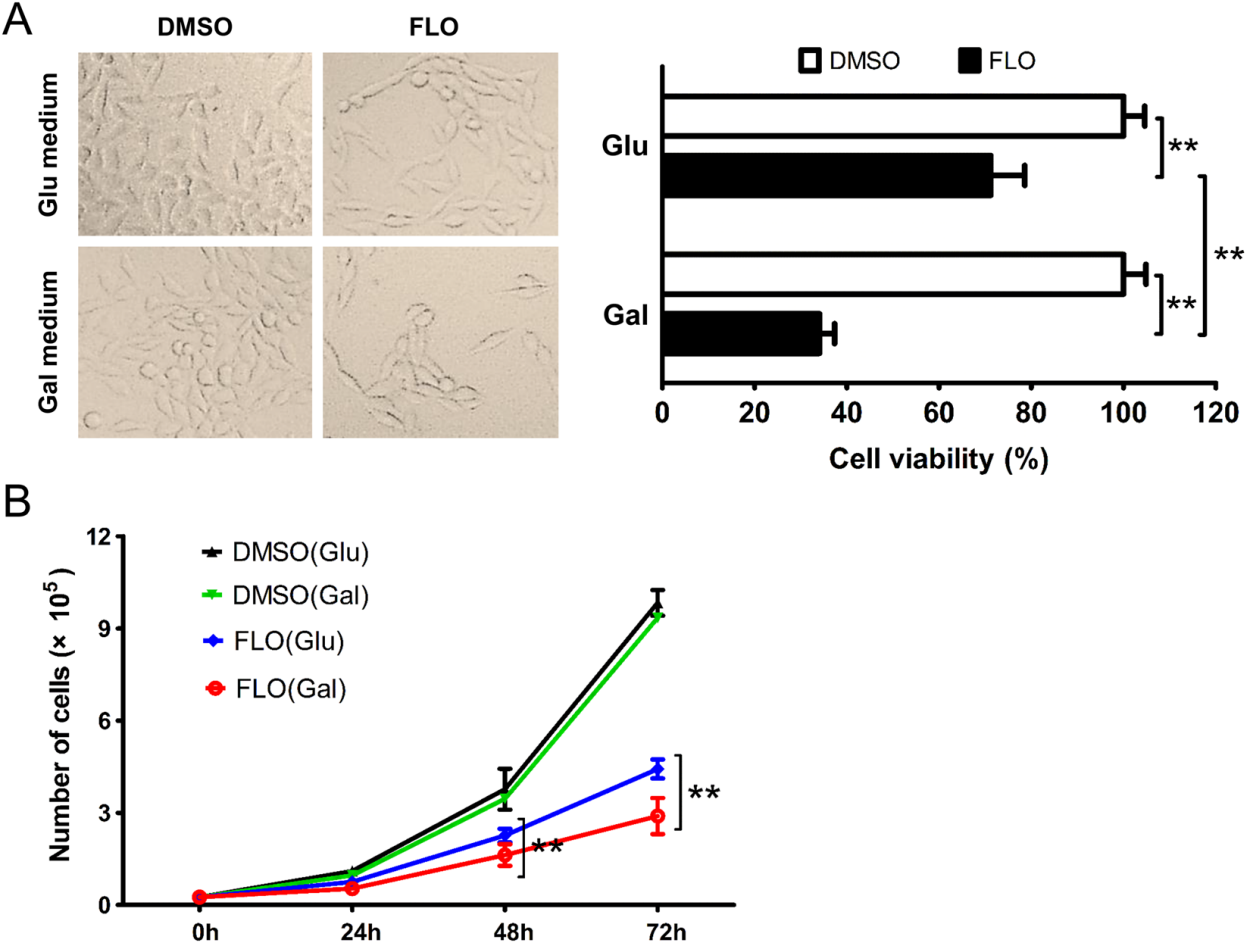
Mitochondrial dysfunction contributes to the decreased cell viability in FLO-treated L cells. (**A**) L cells morphology was observed following the treatment of FLO (0.1 mg/mL) for 48 h. The viabilities of L cells cultured in glucoses (Glu) and galactoses (Gal) DMEM supplemented with FLO (0.1 mg/mL) were detected by using CCK-8 method (n = 5). (**B**) Growth curve of FLO-treated L cells that cultured in glucoses or galactoses DMEM. The results were expressed as the average cell numbers from three independent experiments. **p < 0.01, as determined by LSD multiple comparison tests after one-way ANOVA analysis.

To determine whether FLO’s effects on the viability of L cells are due to mitochondrial malfunction, glucose in the medium was replaced by galactose to avoid *Crabtree effect* ^14^ and force cells to efficiently use mitochondrial respiratory chain *in vitro* ^15^. The results showed that viabilities of mammalian cells including L cells, 293T cells and Hela cells grown in galactose were much lower than that in glucose when supplemented with equal dose of FLO (Fig. 3A,B, Supplementary Figs S3 and S4A), indicating that cell proliferation inhibition caused by FLO treatment was directly related to FLO-induced mitochondrial dysfunction. We further explored the cytotoxicity of some other commonly used antibiotics that worked through inhibiting bacterial peptidyl transferase including chloramphenicol, thiamphenicol, roxithromycin and doxycycline. Similarly, these antibiotics induced different levels of cytotoxicity to L cells when cultured in glucose medium and more severe cytotoxicity was observed when L cells with damaged mitochondrial respiratory chain were grown in galactose medium (Supplementary Fig. S4B). Taken together, these results demonstrate that mitochondrial dysfunction is functionally important for FLO-mediated suppression of cell proliferation and cell cycle arrest.

### Cell death is not involved in FLO-induced cytotoxicity

In addition to the well-established role of the mitochondria in energy metabolism, regulation of cell death has recently emerged as a second major function of these organelles and dysfunctional mitochondria were correlated with the activation and progression of cell apoptosis and necrosis^16^. To further understand the roles of mitochondrial dysfunction in FLO-mediated cell proliferation, we examined that effects of FLO on cell death. Annexin V-PE/7-AAD staining showed that there were no significant differences in apoptosis rates between FLO-treated and untreated L cells after FLO treatment (Supplementary Fig. S5C and D), suggesting that FLO does not affect cell apoptosis. We further analyzed the activity of caspase-3, a member of the cysteine-aspartic acid protease (caspase) family that is activated in the apoptotic cell both by extrinsic (death ligand) and intrinsic (mitochondrial) pathways^17^, and found that FLO-treatment did not change the caspase-3 activity (Supplementary Fig. 5E). Similar results were obtained when cells were analyzed by the release of LDH up to 96h post FLO treatment (Supplementary Fig. 5F). Taken together, these data suggest that FLO treatment does not promotes cell death in L cells.

### FLO activates AMPK and suppresses phosphorylation of mTOR/p70S6K

**Figure 4:**
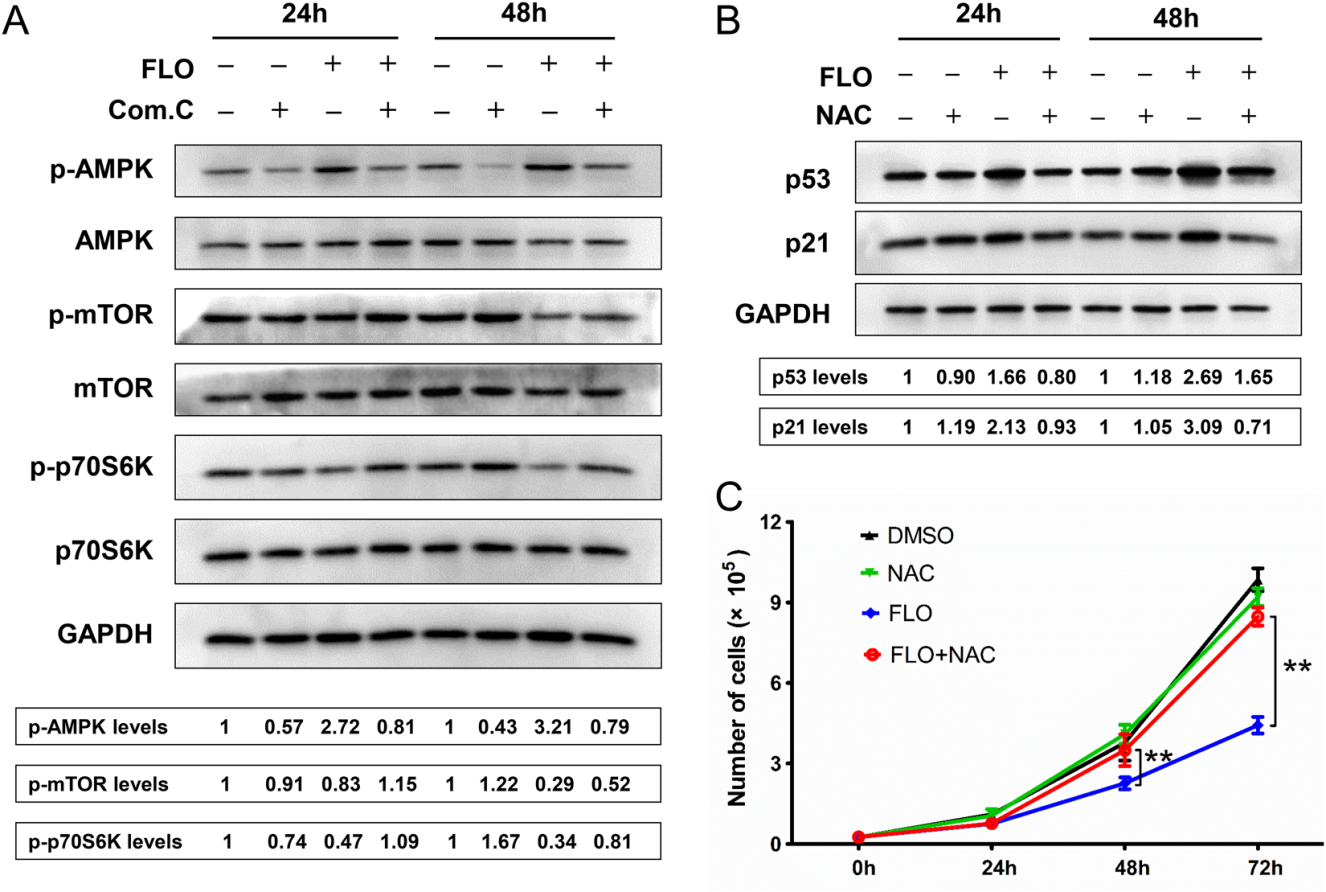
FLO inhibits cell proliferation through suppressing the phosphorylation of p70S6K and elevating p21 levels. (**A**) Immunoblot analysis of the indicated protein expression in L cells treated with FLO (0.1 mg/mL) for 24 h or 48 h. To assess the influences of compound C on the expression of indicated proteins, L cells were pretreated with compound C for 2 h and then treated with FLO for another 24 h or 48 h, respectively. GAPDH served as a loading control. Densitometry was performed for quantification and the ratios of the phosphorylated proteins to GAPDH are presented at the bottom of the blots. Cropped blots are displayed and full-length blots are included in the Supplementary Information file. (**B**) Immunoblot analysis of p53 and p21 protein levels in L cells treated with FLO (0.1 mg/mL) only or co-treated with FLO and NAC (2 mM) for 24 h or 48 h. GAPDH served as a loading control. Densitometry was performed for quantification and the ratios of the proteins to GAPDH are presented at the bottom of the blots. (**C**) The influence of NAC co-treatment on the numbers of FLO-treated (0.1 mg/mL) L cells. The results were expressed as the average cell numbers from three independent experiments. Statistical analysis was performed by LSD multiple comparison tests after one-way ANOVA analysis. **p < 0.01.

As is mentioned above, FLO-treatment decreased the cellular ATP levels and also inhibited cell growth and proliferation (Figs 1H and 2). Given AMPK is intracellular the energy sensor that sensing change of ATP levels, we tested whether the AMPK activation, which is important in regulating cell metabolism and proliferation, was involved in FLO-induced cytotoxicity. Immunoblotting showed that FLO treatment promotes phosphorylation of AMPK at Thr172 site (Fig. 4A). As a result, activation of AMPK inhibited downstream molecules phosphorylation of mTOR and of p70S6K, which are direct downstream molecules of AMPK. Conversely, Compound C, an inhibitor of AMPK, suppressed FLO-induced AMPK phosphorylation and promotes p70S6K activation in L cells (Fig. 4A). Collectively, these results indicate that FLO activated AMPK to inhibit cell proliferation via promoting activated AMPK activation to repress the mTOR/p70S6K pathway.

### FLO promotes ROS generation to activate p53/p21 pathway

As a target of ROS, the tumor suppressor protein p53 serves as a cyclin-dependent kinase inhibitor and can lead to cell proliferation inhibiton^18^. Additionally, p21, one of downstream molecules of the p53 signaling pathway, also plays important roles in cell proliferation and cell senescence through mediating G1-phase cell cycle arrest^19^. After FLO treatment, we found that FLO increased the levels of p53 and p21 (Fig. 4B), indicating that FLO-mediated increase of p53 and p21 may also contribute to the inhibition of cell proliferation. Interestingly, NAC, a scavenger of ROS, could clearly decrease the levels of p53 and p21 compared with the group treated with FLO only, suggesting induction of p53 and p21 by FLO treatment is dependent on ROS level. Consistently, NAC treatment restored the inhibition of cell proliferation by FLO treatment as indicated by an increase in cell viability (Fig. 4C). Taken together, these results indicate that the excessive ROS activates the p53/p21 pathway that contributes to FLO-mediated inhibition of cell proliferation.

### FLO induced autophagy deficiency and senescence-associated β-gal activation

**Figure 5:**
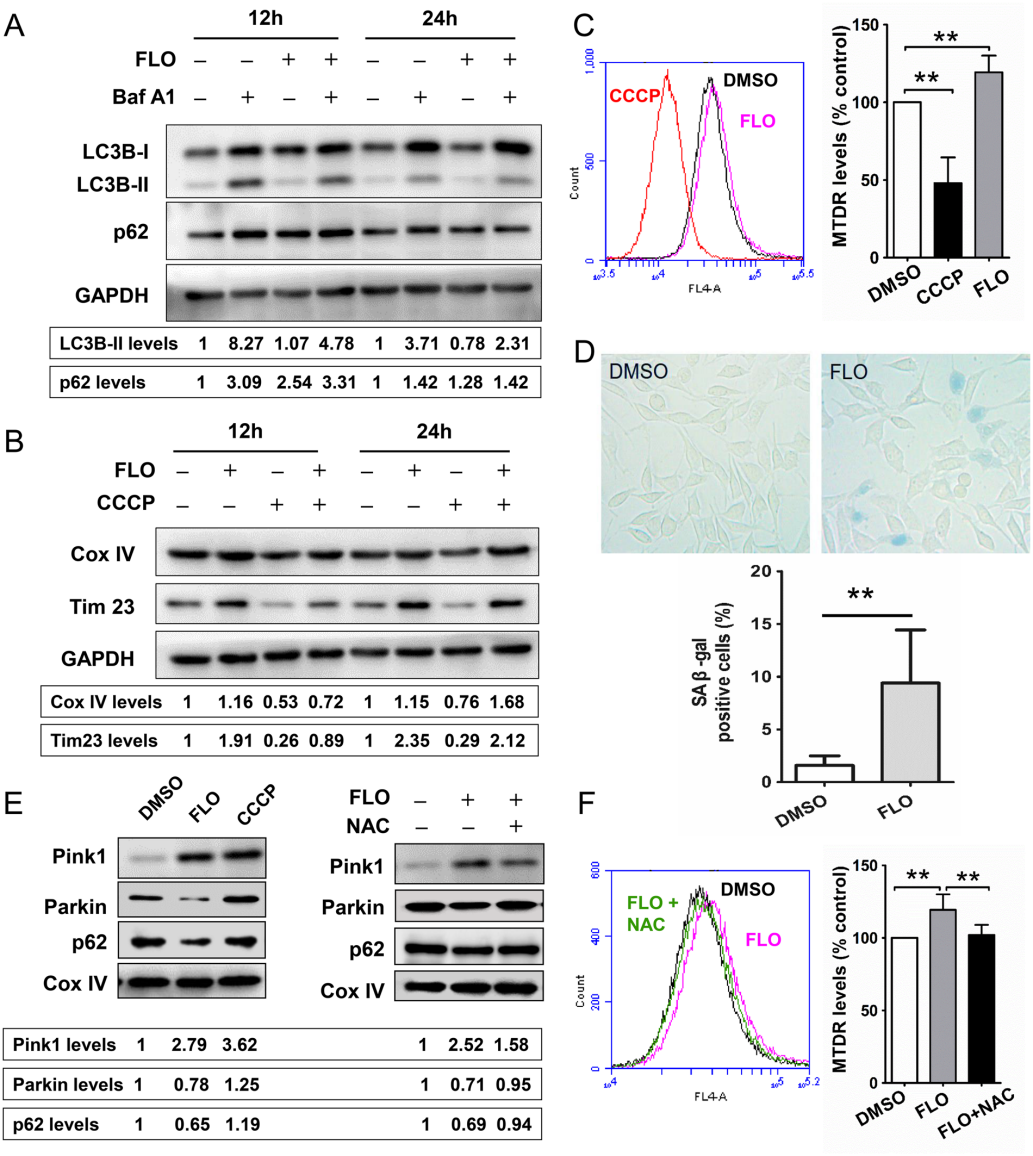
FLO inhibits Parkin-mediated mitochondrial autophagy and promotes cell senescence. (**A**) Immunoblot analysis of autophagy-related proteins in L cells. Cells were treated with 0.1 mg/mL FLO for 12 h or 24 h, and combined treatment with Baf A1 (200 nM) as indicated. GAPDH served as a loading control. Densitometry was performed for quantification and the ratios of LC3B-II or p62 to GAPDH are presented at the bottom of the blots, respectively. Cropped blots are displayed and full-length blots are included in the Supplementary Information file. (**B**) Cox IV and Tim23 levels were detected by western blot with the whole cell lysis extracted form L cells. Cells were treated with FLO for 12 h or 24 h, or were pretreated with FLO for 8 h of 20 h and then co-treated with CCCP (100uM) for another 4 h as indicated. (**C**) Flow cytometric analysis of Mitotracker Deep Red fluorescence in L cells treated with FLO (0.1 mg/mL) for 24 h. The mean fluorescence of Mitotracker Deep Red represents the level of mitochondrial population. CCCP (100 uM for 4 h) was used as a positive control for the clearance of damaged mitochondria through mitophagy. (**D**) Representative images of SA β-gal staining in L cells in the presence of FLO (0.1 mg/mL) for eight days. The medium containing FLO was renewed every two days. SA β-gal-positive cells were quantified (n = 3). (**E**) Immunoblots show levels of the Pink1, recruited-Parkin and p62 on mitochondrial outer membrane in L cells. Cells were treated with FLO (0.1 mg/mL) only or co-treated with FLO and NAC (2 mM) for 24 h. CCCP (100 uM for 4 h) was used as a positive control for the induction of mitophagy through Pink1-Parkin pathway. Cox IV served as a loading control. (**F**) Flow cytometric analysis of Mitotracker Deep Red fluorescence in L cells that co-treated with FLO (0.1 mg/mL) and NAC (2 mM) for 24 h. Histograms in the figure represents the mean ± SD. **p < 0.01, as compared with the indicated group (LSD multiple comparison test after one-way ANOVA analysis).

To determine the impact of mitochondrial quality control on the process of FLO-induced cytotoxicity, we evaluated autophagy in L cells that were or were not treated with FLO. The proteins levels of LC3B and p62 (two crucial markers for autophagy) were assessed by western blot analysis. We also used Bafilomycin A1 (Baf A1), a specific inhibitor of vacuolar type H+-ATPase, which can block the fusion of autophagosomes and lysosomes, to monitor the formation of autophagosomes and autophagic flux^20^. We found FLO-treatment significantly decreased the protein levels of LC3B-II. The expression of LC3B-II was less in FLO and Baf A1 co-treated cells when compared with that in cells treated with Baf A1 only (Fig. 5A and Supplementary Fig. S6). FLO-treatment elevated the levels of p62 (SQSTM1), whose expression level is inversely correlated with autophagic activity and is also used to monitor the autophagic flux^20^. These results indicate that FLO impairs autophagic flux. Analyzing the mitochondrial mass through monitoring the degradation of mitochondrial proteins has been used as a quantitative way to monitor the degradation process of mitophagy^10^. Thus, we detected the levels of Cox IV and Tim23 (a subunit of mitochondrial inner membrane translocase) and found that FLO-treatment could increase Cox IV and Tim23 levels when compared with DMSO-treated cells or mitophagy inducer (CCCP)-treated cells (Fig. 5B). A flow cytometry-based novel method to determine mitophagy by using MitoTracker probe has recently been described for rapid, reproducible, and robust quantification of mitophagic flux^21^. In this study, mitochondrial population levels were further determined by flow cytometry using MitoTracker Deep Red (MTDR) dye. We found FLO-treatment induced a significant increase of MTDR fluorescence levels relative to DMSO-treated cells and CCCP-treated cells (Fig. 5C). Taken together, these results indicate that FLO impairs mitochondrial autophagy and results in damaged mitochondria accumulation in FLO-treated L cells.

Mitochondrial dysfunction and mitophagy deficiency were reported contributed to normal aging and a wide spectrum of age-related diseases. To further study the influence of FLO-induced mitophagy deficiency and damaged mitochondria accumulation on cell fate, the activation of senescence-associated β-gal, a specific biomarker of cell senescence, was detected by the accumulation of blue-violet X-gal crystals in FLO-treated L cells (Fig. 5D). The morphology of these FLO-treated cells was changed to the more flattened and irregular shape characteristic of cell senescence.

### FLO-induced mitophagy deficiency correlated with the inhibition of Parkin recruitment to damaged mitochondria

Recent studies have shown that damaged mitochondria are ubiquitylated by Parkin, an E3 ubiquitin ligase, and then recruited-p62 on mitochondrial outer membrane binds to LC3 to form autophagosome. In this mitophagic process, p62 cooperates with Parkin for the elimination of damaged mitochondria^22^. As is mentioned above, FLO-treatment impaired mitochondrial autophagy and resulted in damaged mitochondria accumulation in the treated cells, thus, we asked whether Parkin recruitment to the damaged mitochondria was affected in FLO-treated cells. Both FLO and CCCP, a well-known mitophagy inducer, increased Pink1 expression, while the levels of Parkin and p62 on mitochondria were significantly decreased in FLO-treated cells (Fig. 5E), indicating that Pink1-Parkin-mediated mitophagy was impaired in FLO-treated L cells. We then treated L cells with FLO combined with the ROS scavenger NAC and found that the recruited Parkin and p62 on mitochondrial outer membrane were elevated when compared with the cells treated with FLO only. In addition, the co-treatment of FLO with NAC decreased the MTDR fluorescence levels relative to that of FLO-treated cells (Fig. 5F), which indicated that NAC-treatment restored mitophagy and promoted the clearance of damaged mitochondria in FLO-treated L cells. These results reveal that FLO-induced mitophagy deficiency correlates with the inhibition of Parkin translocation to mitochondria, and the ROS scavenger NAC can partially restore the impaired mitophagy.

## Discussion

FLO is now widely used not only for therapeutic and prophylactic purposes in veterinary medicine, but also for promoting animal growth, especially in aquaculture^1^. Moreover, residue of FLO in animal products has proved to be a global problem, a clear understanding of the mechanism of FLO-induced cytotoxicity would be benefit to the development of new drugs and to human and animal health worldwide. Here we show that FLO induces mitochondrial damage and ultimately cause cell proliferation inhibition, damaged mitochondria accumulation and cellular senescence.

Mitochondria are eukaryotic cellular organelles specialized for energy conversion and ATP production. Because the mitochondria are originated by endosymbiosis from α-proteobacteria, the mitochondrial ribosomes (mitoribosomes) share more structural similarities and chemical properties to bacterial ribosomes than to eukaryotic cytoplasmic ribosomes^23^. Mitochondrial DNA encodes two rRNAs, 22 tRNAs and 13 proteins that are essential subunits of mitochondrial respiratory chain complexes (I, III and IV). Synthesis of these 13 proteins is performed by mitoribosomes within the mitochondrial matrix. Extensive research has shown that exposure to the antibiotics that belong to aminoglycosides^24^, tetracyclines^25^ and macrolides^26^, whose target sites involve bacterial ribosomes and mitoribosomes, could inhibit mitochondrial protein synthesis and subsequently impair the mitochondrial respiratory chain. In this study, we confirmed that the binding of FLO to the large subunit of mitoribosome could significantly decrease mtDNA-encoded proteins and thus decrease mitochondrial membrane potential and elevate the production of harmful ROS. As consequences of mitochondrial stress, the activities of mitochondrial respiratory chain complex and the generation of cellular ATP were all decreased.

The mitochondrial respiratory chain activity impacts a variety of processes beyond energy balance, such as ROS production^27^, the redox state^28^, mitochondrial protein import^29^, and signaling^30^, since many metabolic pathways, including glycolysis, the TCA cycle, and beta-oxidation, produce the electron donors for fueling the chain. One consequence of respiratory chain dysfunction is impaired cell proliferation, and human cells in culture arrest upon pharmacological or genetic inhibition of complex I or III^31^. In this study, we confirm that FLO-induced mitochondrial dysfunction in L cells caused cell proliferation inhibition in a dose- and time-dependent manner. AMPK is known as a metabolic master switch and is activated by a decrease in the intracellular ATP/AMP ratio. Another important role of AMPK is its participation in the regulation of cell proliferation. Published studies indicate that AMPK activation strongly suppresses cell proliferation in non-malignant cells as well as in tumor cells^32^. Stimulation of AMPK activity requires phosphorylation of the alpha subunit at Thr172 in the activation loop by upstream kinases. The fact that FLO-treatment can induce increasing levels of AMPK phosphorylation at Thr172 supported that FLO induces AMPK activation. Accumulating evidence has revealed that mTOR signaling is one of the major downstream pathways regulated by AMPK, and the activated AMPK suppresses mTOR activity and the phosphorylation of p70S6K activity^32^. The results of this study demonstrated that FLO-induced AMPK activation inhibited the phosphorylation of mTOR and p70S6K. Moreover, co-treatment of FLO with compound C, an AMPK inhibitor, could restore the inhibited mTOR and p70S6K activities and elevate the L cell viability. Based on the decisive role of AMPK/mTOR/p70S6K on cell-cycle and nutrient sensing, in combination with our observation of the inhibitory role of FLO in proliferation, we speculate that FLO is connected to the activation of AMPK/mTOR/p70S6K axis, and AMPK may be, at least in part, one of the appreciated stress-sensing pathways which is involved in FLO-induced cytotoxicity.

The tumor suppressor protein p53 is a potent transcription factor that plays a key role in cell cycle regulation. As a target of ROS, p53 is activated in response to a variety of cellular stress signals and triggers cell cycle arrest to prevent cells from undergoing transformation^18^. Published studies indicate that excessive ROS induced by mitochondrial dysfunction or H_2_O_2_-treatment could significantly inhibits cell proliferation through modulation of cell cycle, accompanied with high expression of p53^33^. In certain cell types, such as in proliferating fibroblasts, induction of p53 by ionizing radiation results in transient arrest in the G1 phase of the cell cycle^34^. In normal diploid fibroblasts, exposure to ionizing radiation results in long term p53-dependent arrest with prolonged induction of the cyclin dependent kinase inhibitor p21, which has been implicated as one major mediator of p53 induced G1 growth arrest^35^. Our present data show that FLO-induced cell cycle arrest and proliferation inhibition in L cells are accompanied with ROS accumulation, p53 expression, and p21 expression, which can be inhibited by NAC, a ROS scavenger. Based on the previous reports and findings from this study, it can be hypothesized that cell proliferation inhibition induced by FLO-treatment might be mediated by AMPK and ROS, cooperatively.

In addition to the well-established role of the mitochondria in energy metabolism, regulation of cell death has recently emerged as a second major function of these organelles. Dysfunctional mitochondria was reported correlated with the activation and progression of cell apoptosis^16^. The inhibition of mtDNA-encoded proteins by nitric oxide and mitochondrial dysfunction induced by acrylamide were reported correlated with the induction of cell apoptosis^36^. While in the present study, we reported that FLO-induced inhibition of mitochondrial protein synthesis and dysfunction of mitochondria in L cells did not trigger apoptosis. Previous study demonstrated that pretreatment of HepG2 and H1299 cells with chloramphenicol, an analogue of FLO, rendered the cells resistant to mitomycin-induced apoptosis^37^. The authors found that chloramphenicol-induced p21 accumulation and redistribution to mitochondria and the inhibition of caspase-3 activation might be important in desensitization to mitomycin-induced apoptosis^38^. While other researchers demonstrated that chloramphenicol inhibited apoptosis in activated T cells by down-regulating the expression of Fas ligand, a type-II transmembrane protein that triggers apoptosis, to make leukemic-like cells more likely to survive long-term and develop into clinical leukemia^39^. Further studies need to illustrate the relationship between FLO-induced mitochondrial damage and apoptosis dysfunction.

The structural and functional changes in mitochondria are implicated in mitochondrial diseases, a clinically heterogeneous group of disorders that characterized by clinical-genetic heterogeneity and frequent multisystemic involvement^40^. The removal of damaged mitochondria through autophagy, a process called mitophagy, is thus critical for maintaining proper cellular functions. Protective mitophagy was reportedly triggered in response to various xenobiotics-induced mitochondrial dysfunction, such as trifloxystrobin-induced keratinocytes toxicity^41^, amitriptyline-induced cytotoxicity^42^, and cadmium-induced nephrotoxicity^43^. Given the fact that FLO-treatment could severely induce mitochondrial damage and decrease the phosphorylation of mTOR, which plays an important role in negatively regulating autophagy, we hypothesized that FLO-treatment could induce mitochondrial autophagy in L cells. Unexpectedly, impaired autophagy in FLO-treated L cells was identified, as demonstrated by a decrease in LC3B-II levels accompanied with an increase in p62 levels. We further analyzed the levels of mitochondrial membrane proteins and mitochondrial mass, the specific indicators of mitophagy, and the results revealed an accumulation of damaged mitochondria in FLO-treated L cells. Consistent with our results, another mitochondrial toxicant doxorubicin, an effective anti-cancer agent that can potentially induce cardiotoxicity, has been shown to impair mitophagy^44^. The elimination of damaged mitochondria in mammals is mediated by a pathway comprised of PTEN-induced putative protein kinase 1 (PINK1) and the E3 ubiquitin ligase Parkin. PINK1 and Parkin accumulate on damaged mitochondria, promote their segregation from the mitochondrial network, and target these organelles for autophagic degradation in a process that requires Parkin-dependent ubiquitination of mitochondrial proteins. This modification recruits the autophagy adaptor molecule p62 and thereby targets mitochondria for autophagic removal^45^. Previous studies revealed that mutations in the RING0 domain of Parkin were important for its redistribution to the damaged mitochondria^46^, moreover, cytosolic p53 could interact with the RING0 domain to disturb Parkin translocation^47^. In the present study, Parkin recruitment from cytosol to mitochondria and subsequent recruitment of p62 were significantly reduced in FLO-treated cells accompanied with the elevation of p53 levels as described above. While co-administrated of FLO with NAC, which could decrease p53 expression through eliminating ROS, could restore mitochondrial recruitment of Parkin and p62, and decrease mitochondrial mass in FLO-treated L cells. Based on the previous reports and our findings, it can be speculated that inhibition of Parkin recruitment to mitochondria mediated by ROS-induced p53 played vital role in the impaired mitophagy in FLO-treated L cells.

It’s well known that TORC1 is one of the most important autophagy regulators. Unexpectedly, low mTOR activity in FLO-treated cells did not initiate autophagy in this study. According to previous reports, it’s not advantageous for cells to initiate autophagy under conditions of moderate glucose limitation and sufficient amino acids. Under such conditions, activation of AMPK should alter cellular metabolism by phosphorylating metabolic enzymes to promote amino acid utilization for energy production. Although AMPK would suppress mTORC1, mTORC1 should not be completely inhibited when amino acids are available. The residual mTORC1 activity may prevent Ulk1 activation, thus minimizing autophagy initiation^48^. Further studies need to illustrate the relationship between FLO-induced mTOR suppression and autophagy deficiency.

Many proliferative cell types like lung and skin human diploid fibroblasts, human melanocytes, endothelial cells, exposed to subcytotoxic stress (UV, H_2_O_2_, ethanol, mitomycin C, hyperoxia, γ-irradiations, homocysteine, hydroxyurea, etc.) undergo stress-induced premature senescence *in vitro* ^49^. Mitochondrial dysfunction and reduction in mitophagy can induce senescence, recently shown to contribute extensively to age-related pathologies, including neurodegenerative diseases^9^ and heart failure^44^. It’s also reported that chloramphenicol and other 70S ribosomal inhibitors such as minocycline, doxycycline and clindamycin could induce p21 induction and senescence-like morphological deterioration^37^. These findings, together with our findings reported here, suggest that FLO-induced p21 elevation and mitophagy deficiency might trigger senescence-associated responses in L cells.

**Figure 6:**
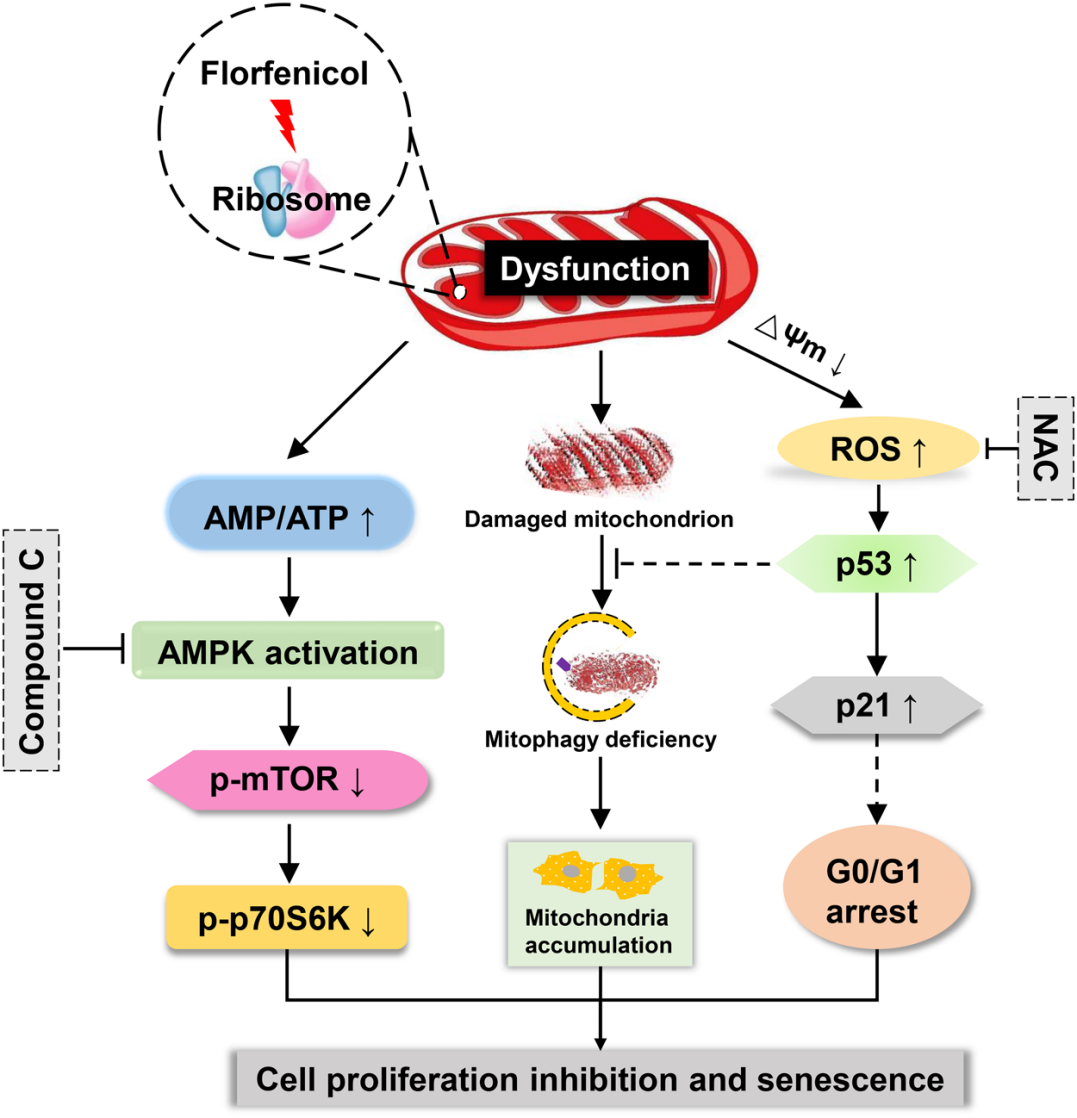
Schematic representation of FLO-induced cytotoxicity in L cells. The exposure of FLO inhibited mitoribosome and lead to mitochondrial dysfunction and structural alterations. Subsequently, cellular ATP biosynthesis was decreased and induced AMPK phosphorylation. The phosphorylated AMPK inhibited the phosphorylation of mTOR and p70S6K, which inhibited cell growth and proliferation. The activation of AMPK/mTOR/p70S6K pathway and inhibition of cell proliferation could be partly reversed by the AMPK inhibitor compound C. The damaged mitochondria also generated more harmful ROS, which elevated the levels of p53 and p21, and contributed to cell cycle arrest and cell proliferation inhibition. The elevation of p53 was also speculated inhibited recruitment of Parkin to the damaged mitochondria and further impaired mitophagy, which caused damaged mitochondria accumulation and induced cellular senescence. The expression of p53, inhibition of Parkin recruitment, accumulation of damaged mitochondria and inhibition of cell proliferation could be partly reversed by the ROS scavenger NAC.

In summary, our results demonstrated that FLO-treatment caused severely structural alterations and dysfunction of mitochondria, and further inhibited cell proliferation by suppressing the phosphorylation of p70S6K and elevating p21 levels through AMPK/mTOR/p70S6K and ROS/p53/p21 pathways, respectively (Fig. 6). Moreover, mitophagy was impaired in FLO-treated L cells which lead to damaged mitochondrial accumulation and cell senescence. This study provides new mechanistic insights into FLO-induced cytotoxicity and may guide proper use of FLO and benefit to the development of safe drugs.

## Materials and Methods

### Reagents

Dulbecco’s modified Eagle’s medium (DMEM) and penicillin-streptomycin solution (100×) were from Gibco (Life Technologies, CA, USA). Fetal calf serum (FBS) was from Biological Industries (Kibbutz Beit-Haemek, Israel). Florfenicol was from Aladdin (Shanghai, China). DMSO, D-galactose and N-acetyl-L-cysteine (NAC) were from Sigma (St. Louis, MO, USA). AMPK inhibitor (compound C) and Bafilomycin A1 (Baf-A1) were from Selleck Chemicals (Houston, TX, USA).

### Cell lines and cell culture

L cells (mouse fibroblast cell line), HEK 293T cells (human embryonic kidney cell line) and Hela cells were obtained from the Cell Bank of the Chinese Academy of Science, and cultured in glucose or galactose-containing DMEM medium, which contain 10% (v/v) FBS, 50 U/mL penicillin and 50 ug/mL streptomycin. Cells were maintained at 37 °C in a humidified incubator with 95% air and 5% CO_2_. Glucose medium and galactose medium were prepared according to previous reports^50^. When the confluence reached to a certain extent, cells will be exposed to different treatment. FLO was dissolved in DMSO with a stock concentration of 800 mg/mL, and was freshly diluted to the desired concentration with culture medium. The final concentration of DMSO was lower than 0.05% (v/v). The details of treatment are stated in figure legends.

### Cell growth curve analysis

L cells were trypsinized and plated in individual wells of 24-well plates for overnight. Cells were then treated with 0.4, 0.1 and 0.025 mg/mL FLO. Every 12 h, the medium was removed, adherent cells were trypsinized and the total number of adherent cells in each well was quantified using Guava easyCyte flow cytometry. The cells counts for 3 wells/time-point were averaged for each group and the data were used to draw growth curves.

### Cell proliferation assay

For CCK-8 cell proliferation assay, cells in an exponential phase of growth were harvested and seeded in 96-well plates. After 12 h culture, the medium was removed and changed to glucose medium or galactose medium supplemented with multiple doses of FLO. The medium was removed and 100uL PBS containing 10uL CCK-8 solution (TransGen Biotech, China) was added into each well and incubated for an additional 1 h and the absorbance was determined using BIO-RAD Model 680 Microplate Reader (California, USA) at 450 nm. The percentage of cell viability was calculated as follows: cell viability (%) = OD_FLO_/OD_DMSO_ × 100%. For EdU cell proliferation assay, cells were grown on coverslips and treated with either DMSO or FLO for 48 h. After treatment, The thymidine analog EdU stock solution (C10310, RiboBio Co., Ltd., China) was diluted and added into the culture medium, and incubated for 2 h. Cells were washed with PBS, fixed in 4% paraformaldehyde, and permeabilized with 0.5% Triton X-100. Apollo^®^ staining solution was added into the wells post washing and incubated for 30 min in the dark. The cells were then counterstained with Hoechst33342 for nuclei. Cells were mounted and examined by using a Nikon microscope with Image-Pro Plus software for image analysis.

### RNA isolation and real-time quantitative PCR

Total RNA was isolated by the acid guanidinium thiocyanate-phenol-chloroform extraction as described previously^3^. The first strand cDNAs were synthesized and quantitative measurements were performed with SYBR-Green PCR Mix. Normalized expression levels were then calculated using the starting levels of GAPDH in each sample to normalize for differences in total RNA content in individual samples. The PCR primer sets for the related genes are listed in the Supplementary information file.

### Flow cytometry

Following treatment with FLO or DMSO for indicated hours, the L cells were harvested using EDTA-free trypsin before or post staining. The apoptosis rates, cell cycle distribution, mitochondrial membrane potential, ROS levels and mitochondrial mass were assessed with the Annexin V-PE/7-AAD staining kit (KeyGen Biotech, Nanjing, China), cell cycle analysis kit (Beyotime, Shanghai, China), JC-1 detection kit (KeyGen Biotech, Nanjing, China), ROS assay kit (Beyotime, Shanghai, China), and MitoTracker Deep Red staining kit (KeyGen Biotech, Nanjing, China), respectively. All flow cytometric measurements were made using a four-colour Accuri^®^ C6 (BD Biosciences, Mountain View, CA). Forward and side scatter light was used to identify target cells based on the size and granularity. 20,000 events were recorded and all data was analyzed using BD Accuri C6 software.

### Biochemical assay

Commercial kits were used to determine mitochondrial complex activities, to measure cellular ATP levels, to detect caspase-3 activation, and to evaluate lactate dehydrogenase (LDH) release according to the manufacturer’s instructions. The kits to determine mitochondrial complex activities were from QIYI Biotech (Shanghai, China), and other kits were from Beyotime Bitotech (Shanghai, China).

### Transmission electron microscope

Cells were harvested, pelleted and fixed in 2.5% ice-cold glutaraldehyde for 12 h, followed by changing the fixative every day until cells were postfixed in 1% osmium tetroxide. After 30 min, the samples were washed with PBS followed by dehydration in a graded series of alcohol and propylene oxide, and then they were embedded in Epon812 Araldite (Electron Microscopy Sciences, Hatfield, PA, USA), sectioned, double stained with uranyl acetate and lead citrate, and analyzed using a transmission electron microscope (JEOL, Tokyo, Japan).

### Immunoblotting analysis

SDS-PAGE and immunoblotting were performed as described^46^. Immune complexes were detected with horseradish peroxidase (HRP)-conjugated second antibody (CWBIO, China) and were visualized by enhanced chemiluminescence (ECL; Pierce). Antibodies used included GAPDH (1:1000, Goodhere Biotech), Cox I (1:300, bs-3953R, Bioss), Cox II (1:1000, 55070-1-AP, Proteintech), ATPase6 (1:1000, 55313-1-AP, Proteintech), Cox IV (1:1000, 11242-1-AP, Proteintech), p-AMPK (1:1000, #2535, CST), AMPK (1:1000, #5832, CST), p-mTOR (1:1000, #5536, CST), mTOR (1:1000, #2983, CST), p-p70S6K (1:1000, #9234, CST), p70S6K (1:1000, #2708, CST), p53 (1:1000, 10442-1-AP, Proteintech), p21 (1:2000, 10355-1-AP, Proteintech), LC3B (1:2000, ab192890, abcam), p62 (1:1000, P0067, Sigma-Aldrich), Tim23 (1:1000, 11123-1-AP, Proteintech), Parkin (1:2000, ab179812, abcam), Pink1 (1:1000, 23274-1-AP, Proteintech).

### SA β-galactosidase staining

L cells were trypsinized and plated in 24-well plates for overnight. After treatments, cells were fixed and stained with β-gal dye (KeyGen Biotech). Cells were observed by using Olympus CX41 microscope to determine the percentage of positive cells (with a blue precipitate) out of the total cells under magnification of ×400.

### Statistical Analysis

Data are shown as the mean ± S.D. Data were analyzed using one-way ANOVA followed by LSD multiple comparison tests by SPSS 20.0 software (SPSS, Chicago, IL). *P* value less than 0.05 was considered statistically significant.

### Data Availability

The datasets generated during and/or analyzed during the current study are available from the corresponding author on reasonable request.

## Electronic supplementary material

Supplementary Information
