# Supplementary material for: Florfenicol-induced Mitochondrial Dysfunction Suppresses Cell Proliferation and Autophagy in Fibroblasts
Source: Sci Rep. 2017 Oct 19;7:13554. doi: 10.1038/s41598-017-13860-9 (PMC5648778; doi:10.1038/s41598-017-13860-9)

**Florfenicol-induced Mitochondrial Dysfunction Suppresses Cell Proliferation and Autophagy in Fibroblasts**

Dongfang Hu^1, 2, 3^, Shengliang Cao^1^, Guihua Zhang^1^, Yihong Xiao^1, 2, 3^, Sidang Liu^1, 2, 3, *^, and Yingli Shang^1, 2, 3, *^

^1^College of Animal Science and Technology, Shandong Agricultural University, Taian Shandong, 271018, China; ^2^Shandong Provincial Key Laboratory of Animal Biotechnology and Disease Control & Prevention, Shandong Agricultural University, Taian Shandong, 271018, China; ^3^Shandong Provincial Engineering Technology Research Center of Animal Disease Control and Prevention, Shandong Agricultural University, Taian Shandong, 271018, China.

^*^ Correspondence and requests for materials should be addressed to S.L. (email: liusid@sdau.edu.cn) or Y.S. (email: shangyl@sdau.edu.cn)

**Supplementary table. The qRT-PCR primer sets for the related genes used in this study.**

| **Gene name** | **Primers (5’→3’)** | **Accession number** |
| --- | --- | --- |
| *GAPDH* | F: CGTGCCGCCTGGAGAAACCTG | BC083065 |
|  | R: AGAGTGGGAGTTGCTGTTGAAGTCG |  |
| *mtCo1 (MTCO1)* | F: ACCACGACGCTACTCAGACTACC | KR020499 |
|  | R: GGAGGGCAGCCATGAAGTCATTC |  |
| *mtCo2* | F: ACCTGGTGAACTACGACTGCTA | AB049357 |
|  | R: CCCTGGTCGGTTTGATGTTACT |  |
| *MTCO2* | F: CGCCATCATCCTAGTCCTCATC | AB626610 |
|  | R: TGAAGATTAGTCCGCCGTAGTC |  |
| *mtAtp6* | F: GCTCACTTGCCCACTTCCTTC | KR020499 |
|  | R: CGGACTGCTAATGCCATTGGT |  |
| *MTATP6* | F: TCGGACTCCTGCCTCACTCAT | AB626610 |
|  | R: GGTGTAGGTGTGCCTTGTGGTA |  |
| *Cox 4 (COX 4)* | F: CGGTGGCAGAATGTTGGCTTCC | NM009941 |
|  | R: GGCAGACAGCATCGTGACATGG |  |


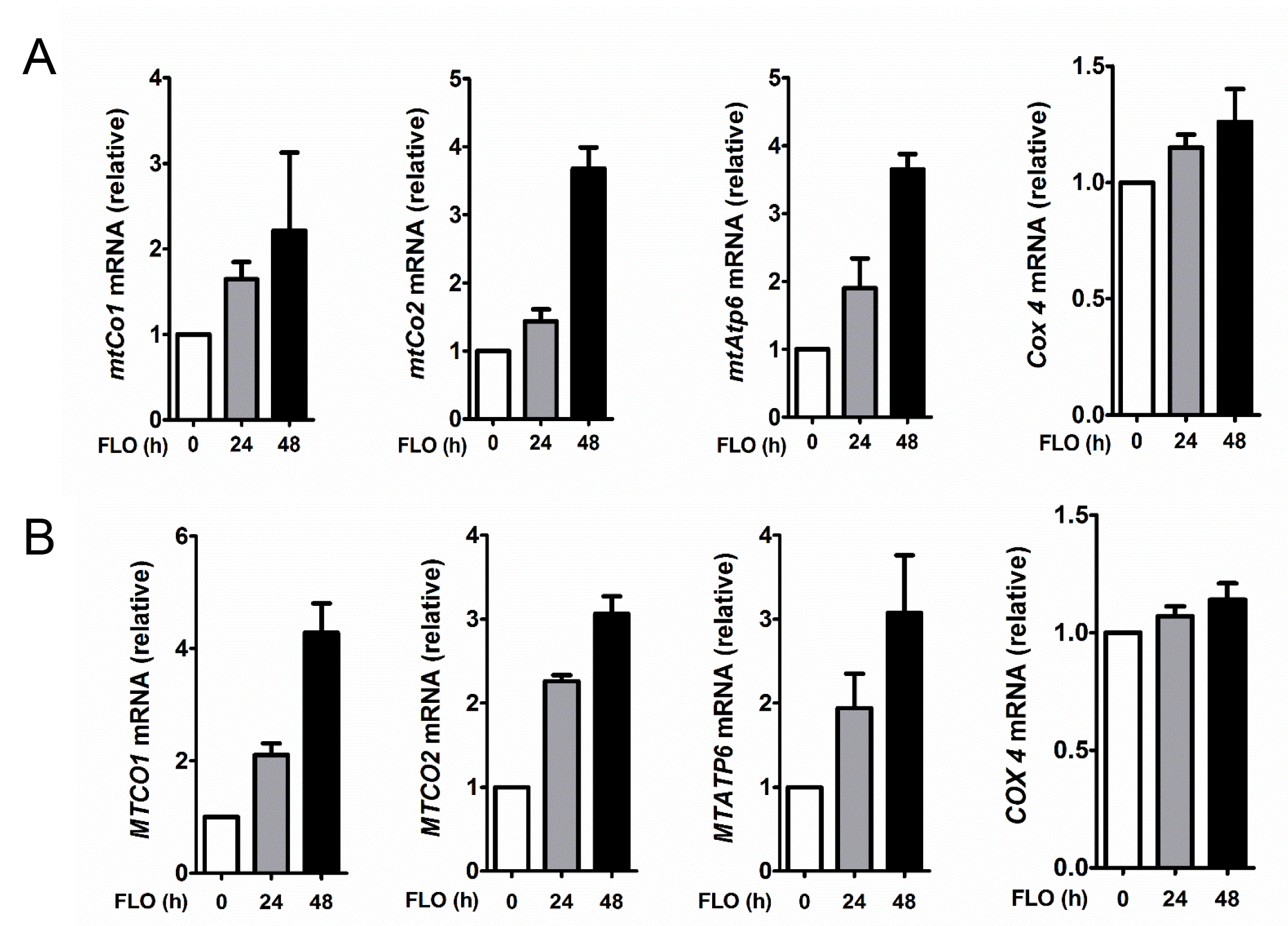


**Supplementary Fig.S1 FLO does not suppress mRNA expression of the mitochondria-encoded proteins.** Quantitative real-time PCR (qPCR) analysis of mRNA expression of Cox I, Cox II, ATPase6 and Cox IV in L cells (A) and HEK 293T cells (B) treated with FLO (0.1 mg/mL) for indicated periods, normalized to expression of the control gene *Gapdh* and presented relative to results obtained for untreated cells. Data shown are mean and s.d. and are representative of two independent experiments. .


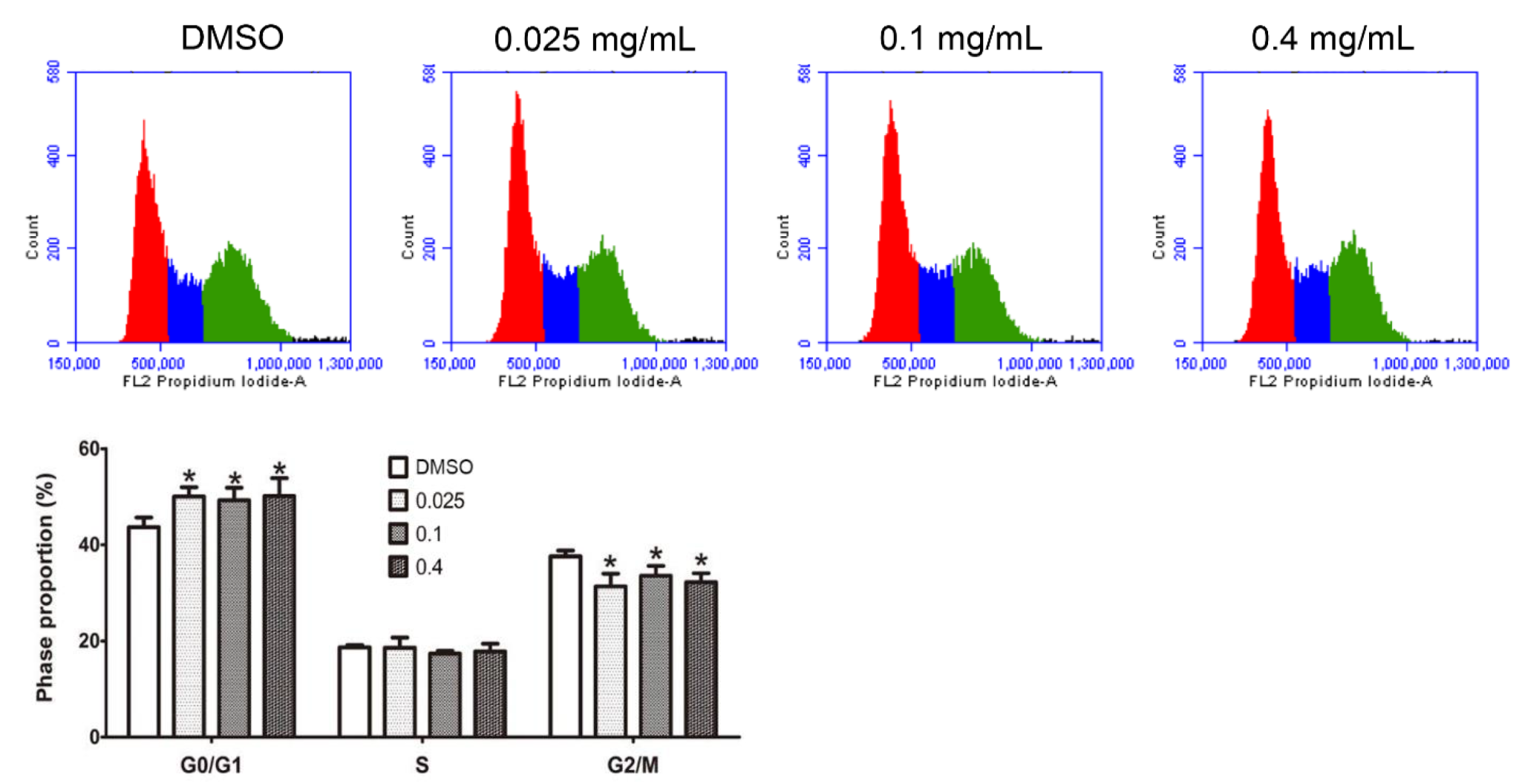


**Supplementary Fig.S2. FLO induces G0/G1 cell cycle arrest in L cells.** Flow cytometric analysis of cell cycle distribution of L cells treated with multiple doses of FLO for 48 h. G0/G1 (Red), S (Blue) and G2/M (Green) phases were demonstrated. Data shown are mean ± SD from three independent experiments. * p<0.05, as determined by LSD multiple comparison tests after one-way ANOVA analysis.


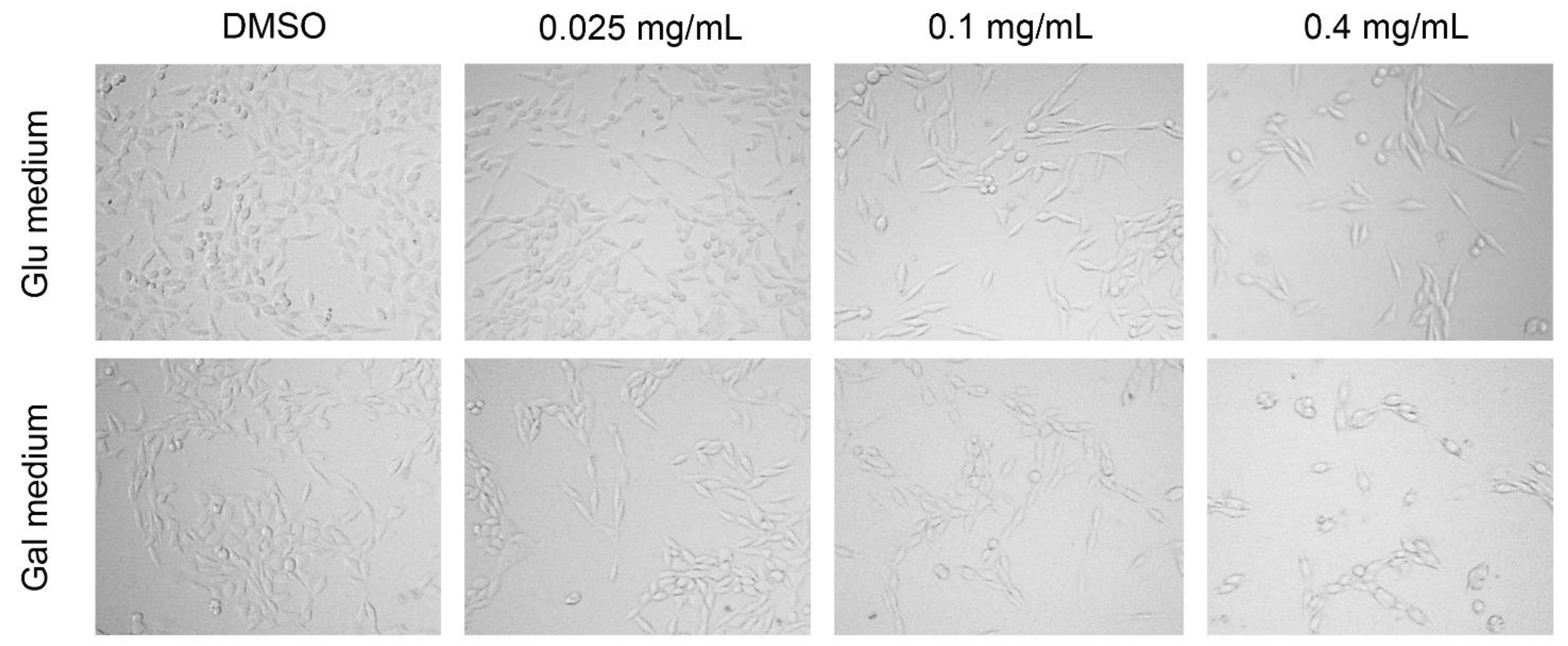


**Supplementary Fig.S3. FLO-induced mitochondrial dysfunction suppresses cell proliferation.** Morphology of L cells treated with various doses of FLO for 48 h in galactose medium or in glucose medium.


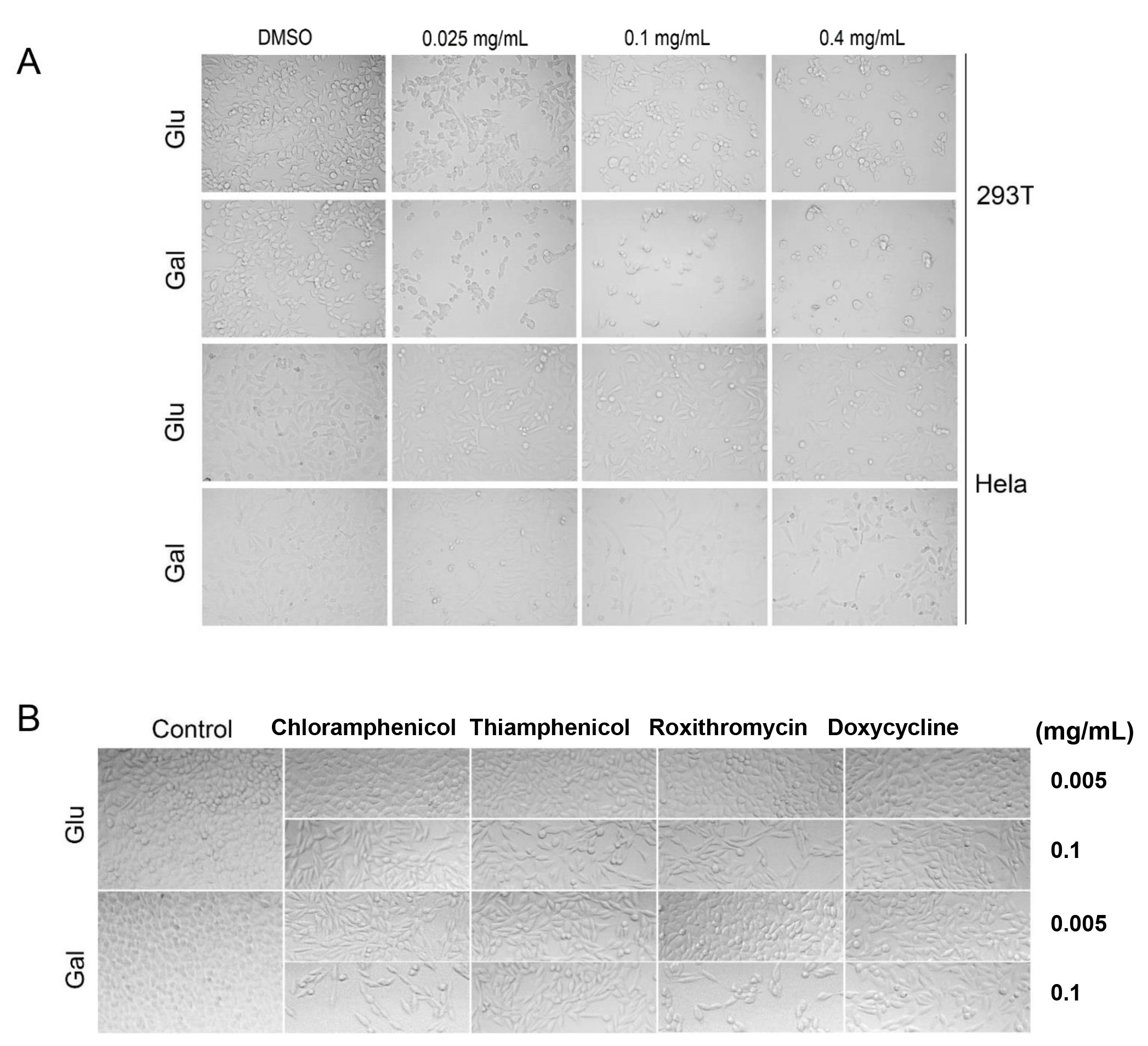


**Supplementary Fig.S4. Mitochondrial dysfunction contributes to drug-induced inhibition of cell proliferation.** (A) Morphology of HEK 293T cells or Hela cells treated of various doses of FLO for 48 h in galactose medium or in glucose medium. (B) Morphology of L cells treated with various doses of multiple antibiotics for 48 h in galactose medium or in glucose medium.


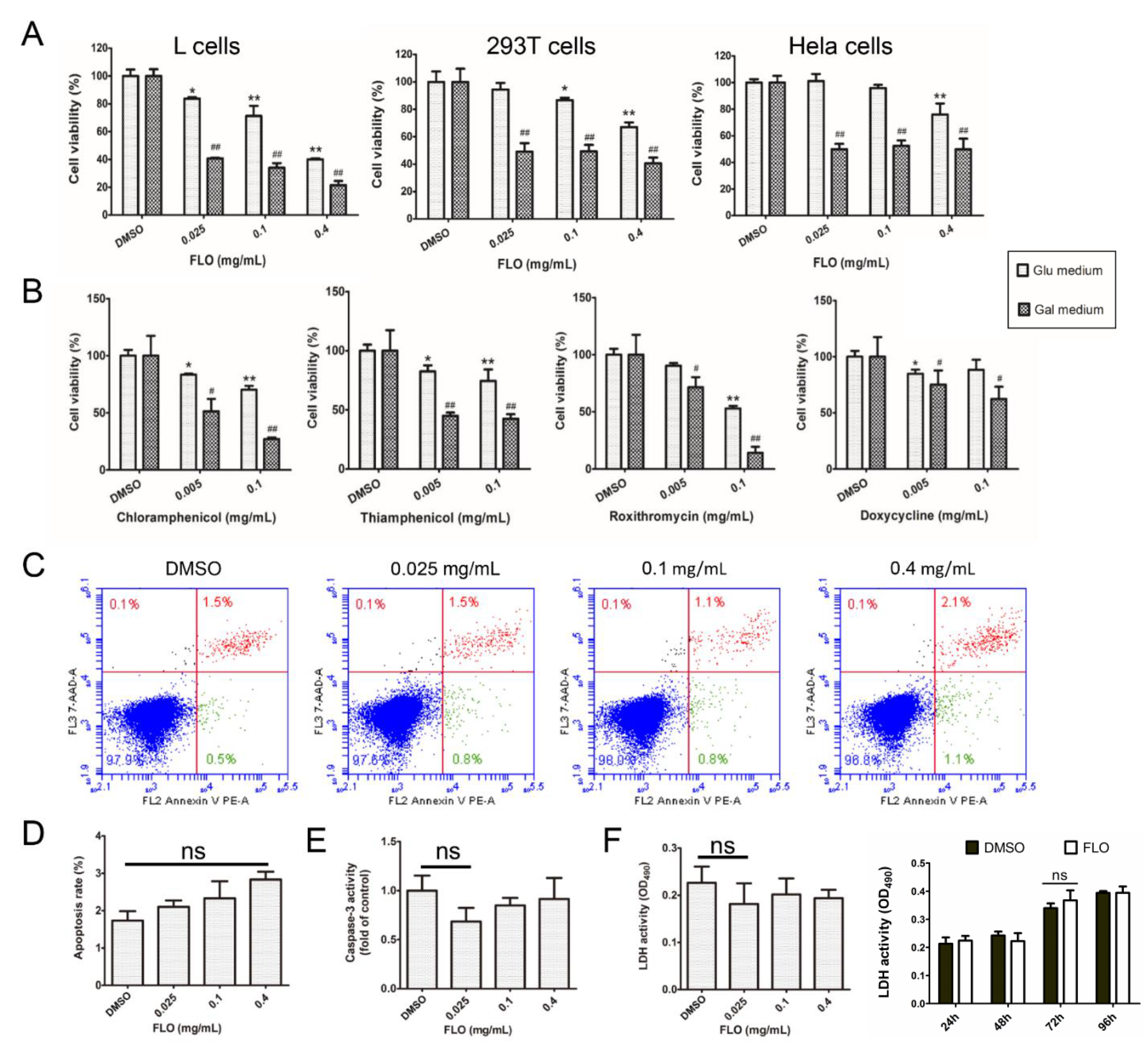


**Supplementary Fig.S5. Florfenicol induces suppression of cell proliferation without affecting cell death.** (A-B) CCK-8 assay to detect viabilities of L cells, HEK 293T cells and Hela cells grown in galactose medium and glucose medium (A) or cell viability of L cells cultured in galactose or glucose medium supplemented with antibiotics that inhibit mitoribosome (B). (C) Flow cytometric analysis of apoptosis of L cells treated with multiple doses of FLO for 48 h by Annexin V and 7-AAD staining. Cells in the right half of the density map were identified as apoptotic cells. (D) Statistics of apoptotic cells as in C. (E) Caspase-3 activity of L cells treated with various doses of FLO for 48 h analyzed by Caspase 3 Assay Kit (Colorimetric). (F) LDH release assay in L cells treated with multiple doses of FLO (left panel) or treated with 0.1 mg/mL FLO for indicated period (right panel). Histograms in the figure represented the mean ± SD. * p<0.05, ** p<0.01, as compared with the control cells cultured in glucose medium. ^#^ p<0.05, ^##^ p<0.01, as compared with the control cells cultured in galactose medium.


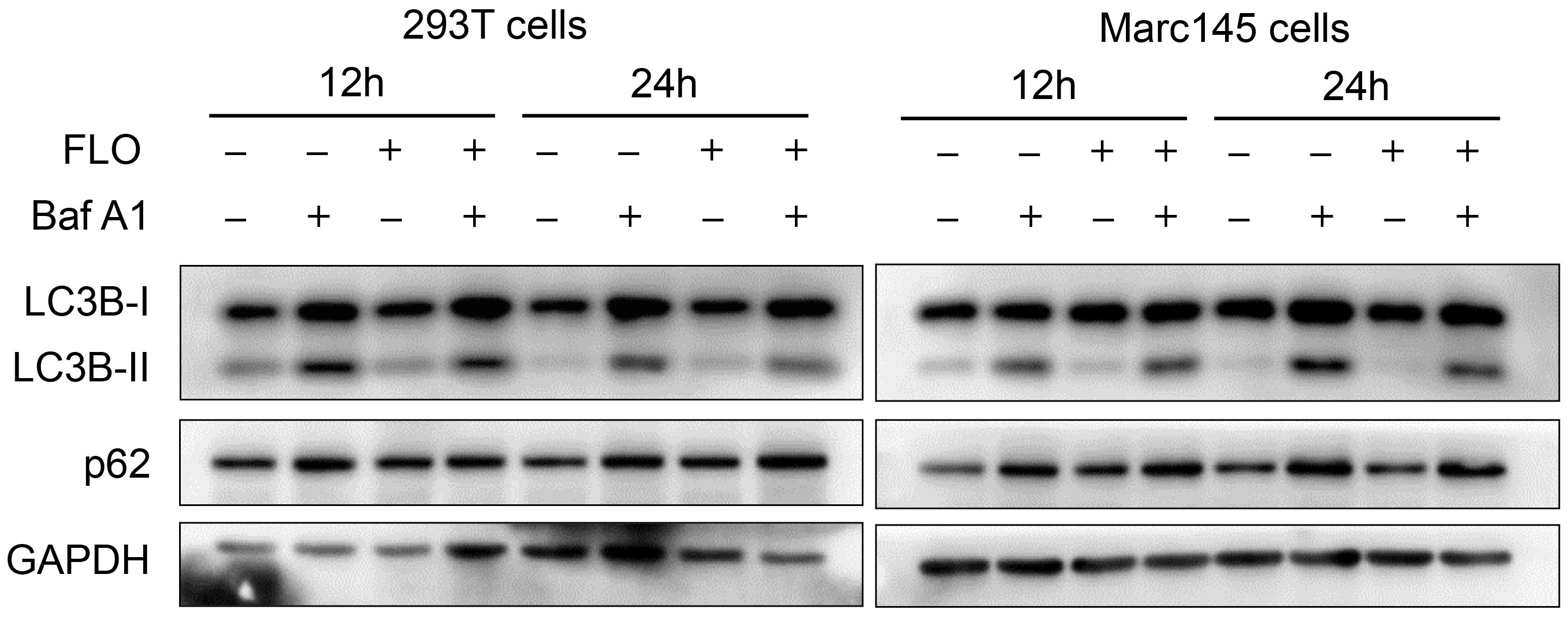


**Supplementary Fig.S6. FLO inhibits autophagy in HEK 293T cells and Marc 145 cells.** Immunoblot analysis of autophagy-related proteins in HEK 293T cells and Marc145 cells treated with Baf A1 (200nM), FLO (0.1 mg/mL) or combination of FLO (0.1 mg/mL) and Baf A1 (200nM) for indicated periods. GAPDH served as a loading control.


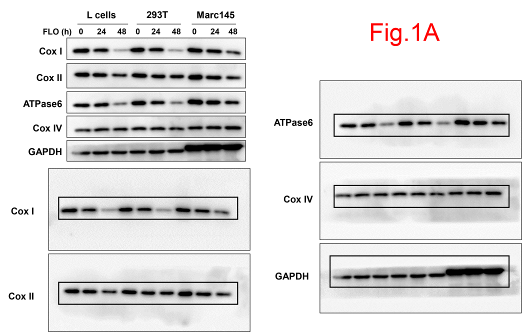


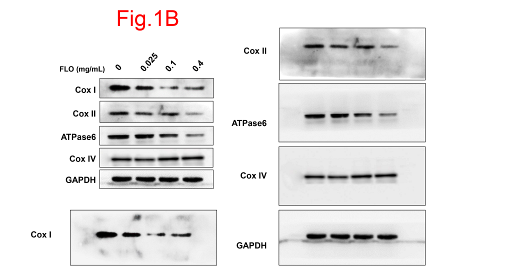


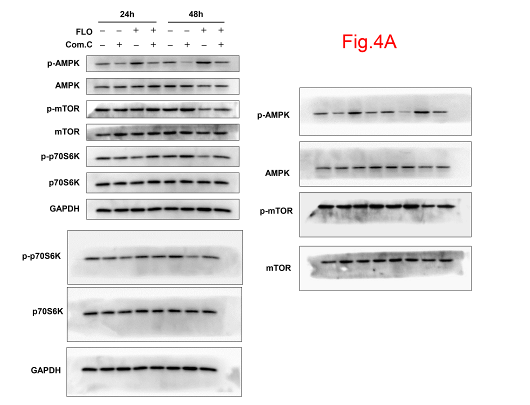


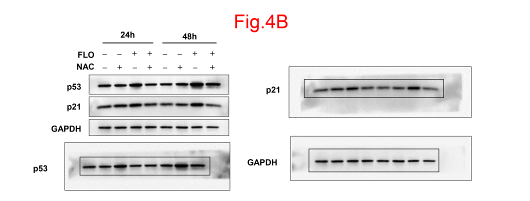


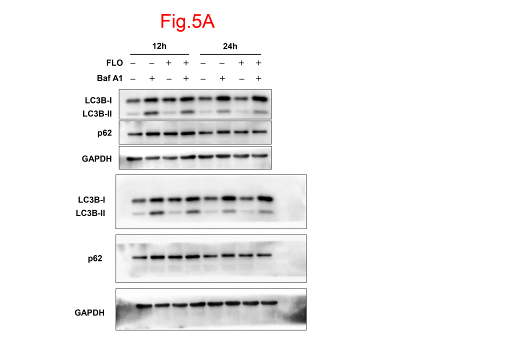

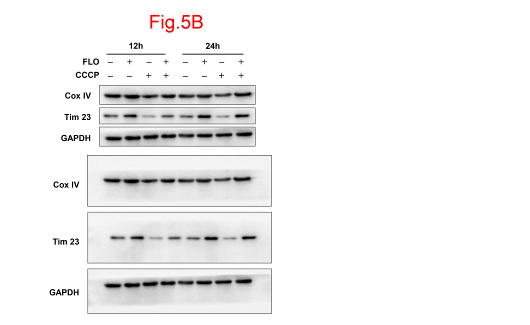


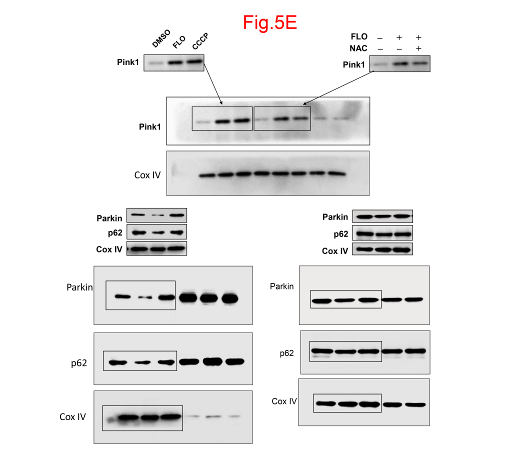

Supplement: Supplementary file 1 — Supplementary Information [file 41598_2017_13860_MOESM1_ESM.doc]
